# Long-Exposure RGB Photography with a Fixed Stand for the Measurement of a Trajectory of a Dynamic Impact Device in Real Scale

**DOI:** 10.3390/s21206818

**Published:** 2021-10-14

**Authors:** Ľudovít Kovanič, Ľubomír Ambriško, Daniela Marasová, Peter Blišťan, Tomáš Kasanický, Michal Cehlár

**Affiliations:** 1 Faculty of Mining, Ecology, Process Control and Geotechnologies, Technical University of Košice, Letná 1/9, 042 00 Košice, Slovakia; lubomir.ambrisko@tuke.sk (Ľ.A.); daniela.marasova@tuke.sk (D.M.); peter.blistan@tuke.sk (P.B.); michal.cehlar@tuke.sk (M.C.); 2 Institute of Informatics, Slovak Academy of Sciences, Dubravska cesta 9, 845 07 Bratislava, Slovakia; tomas.kasanicky@savba.sk

**Keywords:** conveyor belt, long-exposure RGB photography, measurement of trajectory, dynamic impact analysis

## Abstract

The present manuscript proposes a novel method for the measurement of a trajectory of a falling impact hammer in the dynamic loading of conveyor belts and the determination of their impact resistance. The proposed method has been experimentally tested and the results of the measurements are presented in this manuscript. The proposed method is based on the long-exposure photography with a long-duration opened shutter of the Nikon D5000 DSLR camera. Results of the experimental research were compared with direct reference measurements performed using the L-GAGE LT3 laser distance sensor. Differences between values, obtained by the new method and by the reference measurements were up to ±3 mm. The standard deviation identified in all the experiments was 1 mm.

## 1. Introduction

A conveyor belt is a rubber composite material consisting of various materials, such as rubber, fabric, steel, and other materials [1]. It is the most expensive structural component of every belt conveyor, representing 40–60% of the total cost [2]. Conveyor belts have a simple structure; they are universal, flexible, and suitable for transporting bulk materials for short, moderate, and long distances [3]. Depending on the material of a cover layer and a carcass, conveyor belts may be divided into (i) rubber–textile conveyor belts, (ii) plastic conveyor belts, (iii) nylon conveyor belts, and (iv) steel cord conveyor belts [4]. Compared to steel cord conveyor belts, rubber–textile conveyor belts are used more frequently due to their advantages, such as lower costs and simpler maintenance [5].

A conveyor system of multiple conveyors contains the sections between two adjacent members of a conveyance line where the transported material is passed from one line member to another [6]. At these sections (chutes), material particles change their direction and speed; as a result, conveyor belts wear due to abrasion and suffer punctures by larger pieces of the material [7]. Conveyor belts are usually used for continuous transport of sharp-edged materials, which consequently cause abrasive wear of a rubber cover layer, a carcass tear, or disconnection of joints in a rubber–textile conveyor belt [8]. The impact of transported materials on conveyor belts often causes damage classified as a puncture [9]. This type of damage also affects the top cover layer, the carcass and the bottom cover layer of a conveyor belt [10]. The utilisation of a belt is accompanied by negative phenomena which may significantly reduce its service life. Potential methods for reducing such negative effects, mainly by reducing the number of chutes, were described in a paper [11]. Proper operation of a conveyor system consisting of rubber conveyor belts requires high-quality conveyor belts with suitable material composition and the physical and mechanical properties appropriate for the given operating conditions [12]. The costs of procurement, maintenance, general repairs, and restoration of a conveyor belt exceed the maintenance costs of other structural parts of a belt conveyor. Therefore, it is necessary to reduce such costs by extending the service life of conveyor belts [13], which are the components with the highest failure probability [14].

As belts are permanently exposed to loading during their operation, it is necessary to understand their characteristics at various levels of wear or damage. Such factors cannot be verified during the operation; that is why laboratory (simulated) tests of conveyor belts should be conducted [15]. Thus far, several laboratory testing stands have been manufactured; they are based on different principles to examine various parameters. A testing device for testing the conveyor belt puncture resistance, which is available at the Wroclaw University of Technology, has a range for measuring the heights of a falling hammer [16]. Another stand, which is used in a conveyor belt laboratory of the Technical University of Košice, uses a laser distance sensor to measure a falling hammer’s trajectory. This laser sensor continually measures the distance between the hammer and the conveyor belt and has been used in a patent [17]. In testing the impact resistance of conveyor belts, it is essential to measure a drop height of an impactor. It is a critical factor that affects the degree of conveyor belt damage. A trajectory of a falling hammer can be recorded using many techniques. For this purpose, in drop weight testers, which are available on the market, a drop height program was used, controlled by a motor drive with the automatic lifting of the drop weight with stepless adjustment [18]. The issues regarding a path identification of a moving load based on multiobjective optimization were discussed in a paper [19].

Authors Boracchi et al. [20] proposed a model used to reconstruct a falling ball trajectory from a single long exposure motion-blurred image in the field of sports. Wu et al. [21] presented a novel and interesting technique for measuring coal particles’ velocity and diameter. They used a laser beam and a long exposure image to investigate the speed and size of coal particles. They measured small particles of coal dust-sized in the order of 100 µm. The exposure time in these experiments ranged from 600 µs to 3200 µs. Finally, Rengaswamy et al. [22] applied long-exposure photography to measure astronomic phenomena. The exposure time ranged from 0.7 s to 1 s. To increase the details in the images, they used several overlapping images obtained gradually in the intervals of 10 to 20 s. In our investigation, we could not apply such a procedure due to the short duration of the impact hammer motion and as in each measurement, a motion trajectory was different.

Caglioti et al. [23] evaluated the motion of a table tennis ball from a blurred image. They assumed that the movement speed was 10 m/s, a distance between the ball and the camera ranged from 30 to 50 cm, and exposure times ranged from 1/10 to 1 s. Edges were detected using the Canny edge detector [24] and their own approach to detect motion trail edges in an image. In their experiments, a light object was moving in front of a dark background. These approaches could not be applied in our investigation as, in our case, the target trajectory was vertical, and the target crossed the same place several times. That is why we chose the manual approach. This article describes a novel method for the measurement of a falling hammer trajectory. The proposed method has been experimentally tested, and this paper presents the test results. The proposed method is based on long-exposure photography. Long-exposure photography uses a long-duration shutter speed. A trajectory of an impact hammer indicates its motion in a single image during a long exposure time (lasting several seconds).

The inspiration for our novel approach to measure the trajectory of a fast-moving, falling, and bouncing object also came from the geodetic (surveying) principles in previous researches. Geodesy has several surveying techniques and devices that can also be classified as contact and contactless. Contact methods are generally more accurate, but take a longer time to survey [25]. Distance meters in total stations commonly determine the distance with a resolution of 0.1 mm [26]. However, the limiting factor is also the necessity of the static position of the measured object, which is not possible in the case of a falling device. The non-contact method of terrestrial laser scanning (TLS) was used to document the spatial parameters of static objects [27,28,29]. It was also successfully applied to determine the deformation of machinery-rotary kiln during its shutdown [30], but we also successfully verified it with a slow continuous rotary motion of the kiln (1.5 rpm). Deployment of TLS was, therefore, not appropriate for our current research. The fast development of digital photogrammetry with the SfM approach opens up many possibilities for documenting spatial objects and their changes with high accuracy under certain conditions higher than TLS. The problem is the longer time required for one photogrammetric imaging (several minutes) [31,32], which disqualifies this method from measuring the trajectory of the falling impactor. The advantage could be the completeness of the static photogrammetric model [33,34,35,36].

## 2. Materials and Methods

The subjects of our investigation included conveyor belts and other rubber composites, for which it is necessary to identify their impact resistance when exposed to dynamic loading. The aim is to determine an optimal drop height at chutes, and an optimal trajectory for achieving that material falls onto the centre of a conveyor belt without damaging it.

### 2.1. Analysis of the Material Impact

**Figure 1:**
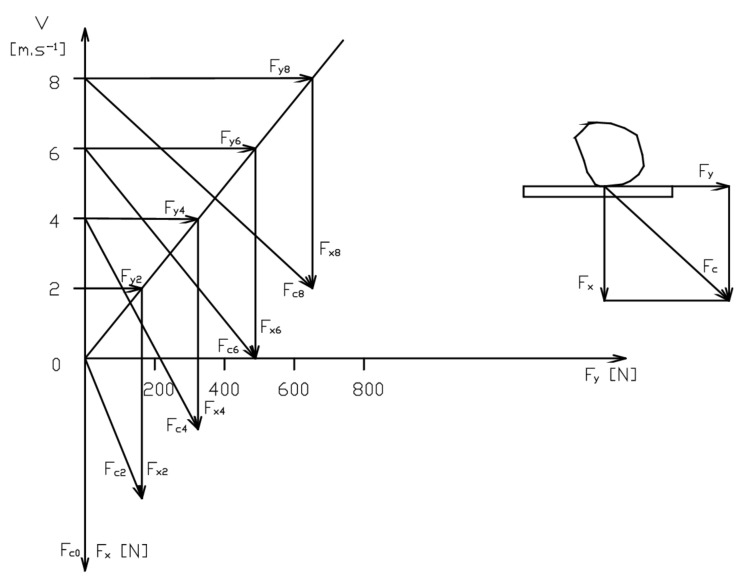
A course of the impact force after an object falls onto a conveyor belt, as a function of the conveyor belt speed and inclination height.

One of the main reasons why conveyor belts are damaged is the point impact loading. An elastic-plastic response follows an impact of a material with a certain weight and speed. Value that characterises the course of such an impact is the impulse of the impact force, and its magnitude and direction are primarily affected by the chute design. Thus, when a material, especially pieces of material, falls down perpendicularly onto a conveyor belt, it induces strong impact forces, which result in punctures of the belt. The intensity of such dynamic effects may be seen in Figure 1, which shows how the resulting impact force *F_C_* increases as the speed of the conveyor belt and an inclination angle increase.

The risk of puncture most frequently occurred with force *F_C8_*, while with *F_C0_* there was almost no risk. This force causes elastic stamping of the cover layer. In addition, as the conveyor belt is in motion, the falling material receives a strong horizontal impulse once it physically contacts the belt. 

### 2.2. Experimental Test Stand

**Figure 2:**
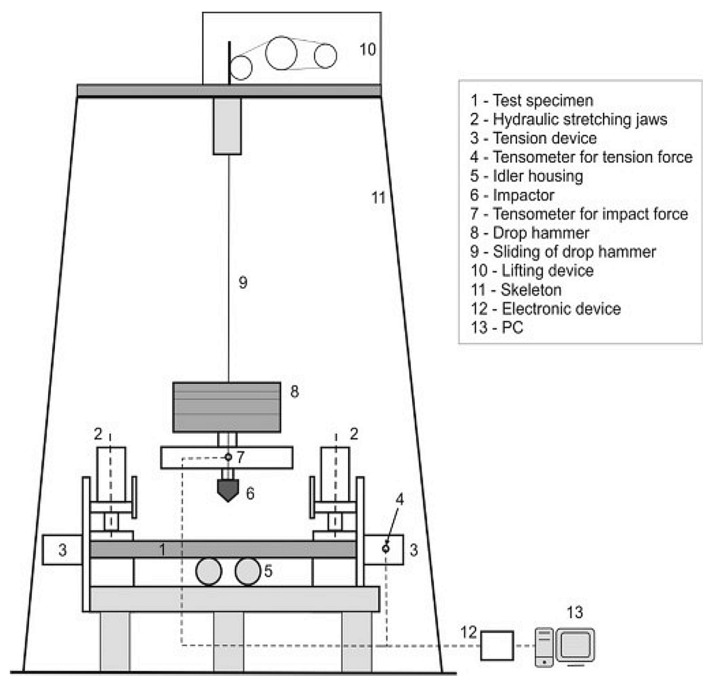
Testing stand scheme.

The experiments were carried out in the Laboratory for Conveyance System Components at the Institute of Logistics and Transport of the Technical University of Košice, where a testing stand was designed and constructed a few years ago for puncture testing of conveyor belts (Figure 2) [10].

**Figure 3:**
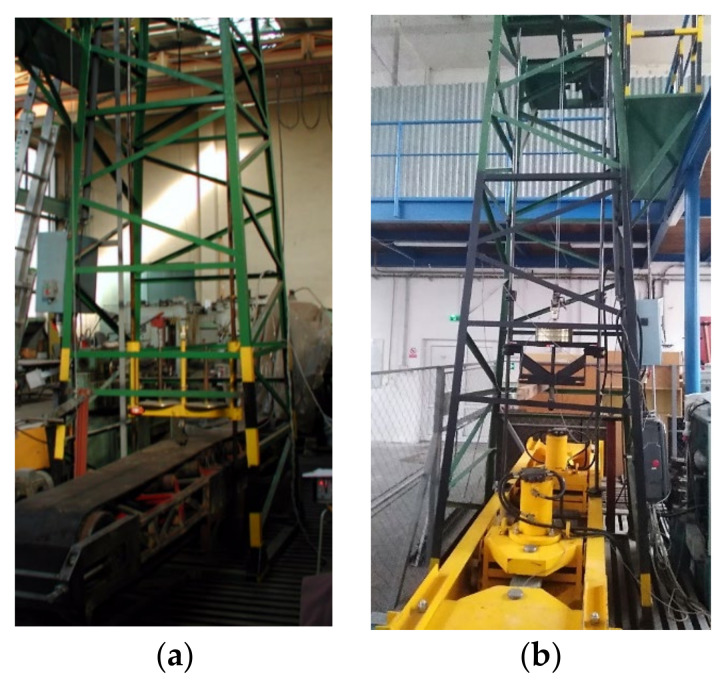
Testing stand; (**a**) original, (**b**) innovated.

In 2006, the testing stand (Figure 3a) was innovated [37] to reduce a length of a test specimen from 10 m to 1.5 m. Another purpose of such innovation was to facilitate a more accurate setting of the tension force applied to a conveyor belt. Before the innovation, belts were rigid-stretched using a screw. That is why hydraulic jaws were installed on the stand to facilitate attaching and stretching a belt specimen by applying a controllable force. These jaws replaced the previously used belt conveyor (Figure 3b).

### 2.3. Experimental Methodology

**Figure 4:**
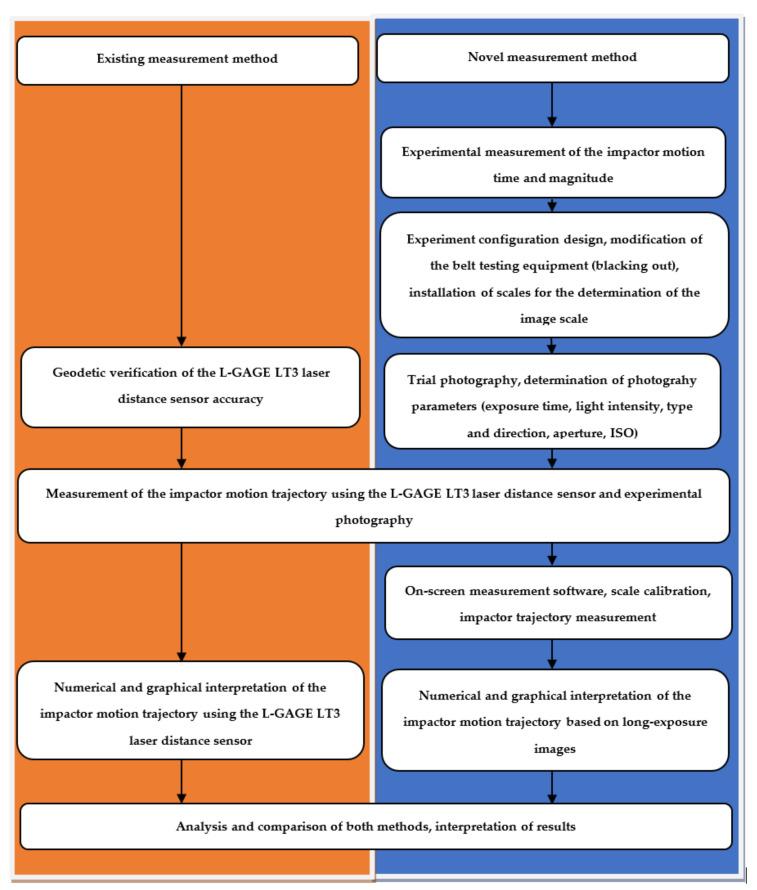
Workflow scheme.

Experimental measurements were carried out as presented in the following workflow scheme (Figure 4).

The measurements of the impactor motion trajectory were carried out using the L-GAGE laser distance sensor and experimental photography. The camera aperture consisted of thin crescent-shaped blades which overlap to form a “circular” opening in the centre. The intensity of the light coming through the lens depends not only on the size of this opening but also on the lens’s focal length. The parameter specified for lenses is an f-number, which is subject to the following equation:

**k = f/D,** (1)

where **k** is the **f**-number; **f** is the focal length (mm); and **D** is the diameter of the entrance pupil of the lens or the aperture opening (mm).

The exposure time is also referred to as the shutter speed. It represents the time of exposure of photosensitive material to the effects of the light coming through the lens.

The aperture, exposure time, and ISO sensitivity together form an exposure triangle that affects the resulting image. An initial image may be corrected in post-processing by adjusting the brightness values using a histogram. Typically, the purpose is to achieve a homogeneous development of histogram values and optimal image balancing. In our experiment, we did the opposite—the resulting images were significantly under-exposed to make the impactor path trail as visible as possible in an image while the surrounding objects were less visible.

During testing, an impact hammer with a weight of 105 kg and a pyramidal impactor (with a base sized 50 × 50 mm) was lifted by a pulley to a predetermined height. Then, it was dropped in a free-fall onto a stretched rubber–textile conveyor belt. Two tensometric sensors were used to measure a magnitude of the tension force FT (accuracy of ±500 N) and a magnitude of the impact force FI (accuracy of ±30 N). During all experiments, the force values were measured. The tension force ranges from 30 to 42 kN, and the impact force’s magnitude ranges from 14 to 30 kN. The height of the impactor ranges from 1089 to 1122 mm. 

**Figure 5:**
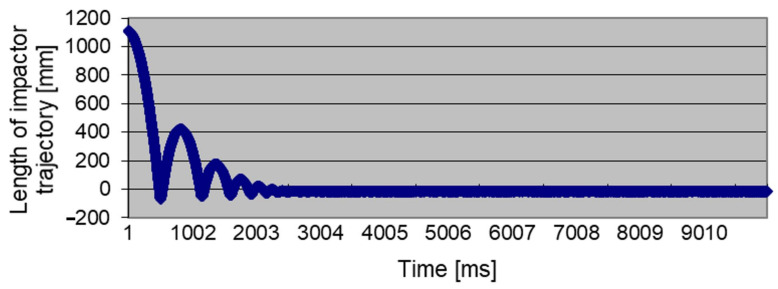
Time series of the measured height of the impact hammer—records from the L-GAGE LT3 distance sensor.

A baseline height of the impact hammer in a resting position and the heights of the impact hammer motion turning points h (trajectory) were measured using the L-GAGE LT3 Long-Range Time-of-Flight Laser Sensor. Distance meter measures lengths ranging from 0.3 to 5 m while using a 90% white card. This distance sensor facilitated measurements in high-frequency images and was installed on the upper part of the testing stand in a stationary position. During the whole movement, the measured data were recorded using the PP065 electronics, which comprised a programmable control panel with analogue and digital inputs and outputs and a serial communication channel, which may be used not only as a transducer, but also as a small control panel. The output of the software that processed the measured values was a text file containing the time t (ms), an impact hammer height h (mm), a magnitude of the tension force FT (kN), and a magnitude of the impact force FI (kN), where each line represented the values recorded at a particular time point. In a single 10-s measurement cycle, 10,000 values were recorded at 1000 Hz for each parameter listed above. Subsequently, the measured data were used to identify the time series of the measured parameters (Figure 5). The impact hammer weight was 50 kg without additional calibrated weights, while the measurements were carried out with the maximum weight. The maximum drop height of the impact hammer was 2.6 m. Considering the fields of view of the used cameras, the drop height was determined as 1 m.

#### 2.3.1. Experimental Method and Setup Description

**Figure 6:**
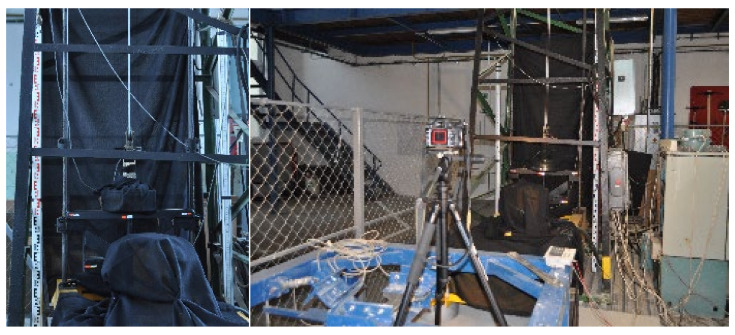
Experimental setup.

In the experimental setup, reflection marks were placed on the body of the impact hammer. The whole body of the impact hammer was covered with black fabric to avoid undesired reflections of its constructional parts. A reference scale was formed of two compared geodetic levelling rods, one with a centimetre scale and the other with a millimetre scale. They were placed in a vertical plane crossing the guide bars of the impact hammer to ensure that the respective measurements correspond to the measurement plane in the monitored points. Undesired reflections in the images were eliminated by applying a matt coating onto the impact hammer (Figure 4) and covering the glazing and bright surfaces with a matt black fabric (Figure 6).

Dimensions of the fixed parts of the impact hammer and distances between the stuck reflection marks were also measured. The reflection marks represented the key elements of the experimental measurements as, as they moved at long exposure, they left continuous trails, which were subsequently evaluated. A Nikon D5000 DSLR camera was placed on a tripod facing the hammer. Its distance from the impact hammer was determined so that the visual field of a single image contained, without zooming, all the setup elements essential for the measurement, i.e., the levelling rods and the impact hammer with the targets, within the entire expected range of motion. The initial camera zooming was carried out using autofocus at artificial illumination. During the measurements, the zooming was manually set to a fixed constant value determined by the autofocus. The camera’s position was not changed after the zooming was set. All the measurements were carried out after the sunset, i.e., without natural light coming through the windows. Such conditions facilitated obtaining almost a stable environment; as a result, the camera settings were constant in all the measurements. Thanks to a dark environment, no ND filters were needed during the photography. The scene was illuminated by a 5000 lm handy led light source placed near the camera lens to ensure that the light would reflect from the reflection marks to the cameras. A diagram of the experimental setup is shown in Figure 6 on the right. A shutter speed was determined experimentally so that the entire experiment (impact hammer motion) could be captured. Its value was determined as 4 s. During the trial photography, an appropriate aperture was gradually identified and set. Hence, the exposure setting procedure was in contrast to the standard procedure, in which the time is adjusted to the determined aperture.

**Figure 7:**
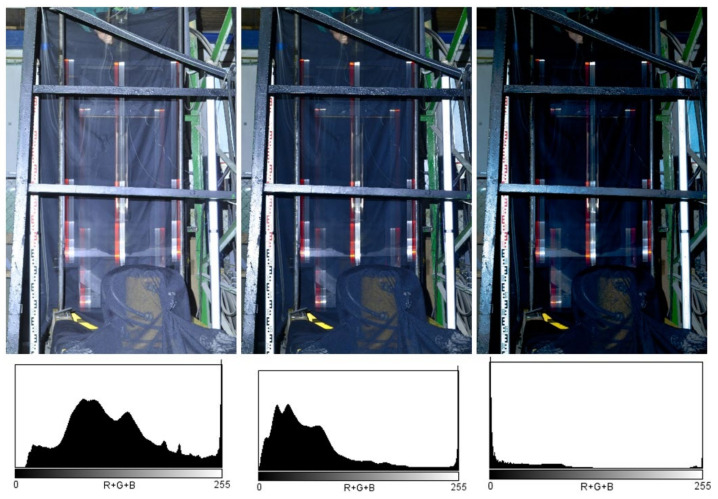
Images of the trajectory of the falling impact hammer (the original on the right; adjusted images in the middle and on the right).

The camera setup parameters were set to a shutter speed of 4 s, aperture f/8 and ISO 100. A raw format was chosen due to expected post-processing adjustments of images. The photography was made using a time-delay shutter release without a photographer’s contact to avoid undesired blur in images caused by the camera motion. Shutter opening was followed by a manual release of the impact hammer. An example of the obtained image is shown in Figure 7 on the left. It shows the impact hammer’s motion trajectory. Orange and white reflection targets formed continuous trails that corresponded to the impact hammer motion. The turning points of the vertical movement of the impact hammer are well visible thanks to a prolonged exposure time during the photography. The images were additionally processed by moderate or significant underexposure to increase the visibility of the scale-identifying elements and turning points, as shown in Figure 7 in the middle and on the right. The histograms express the exposure parameters after the images were adjusted. 

**Figure 8:**
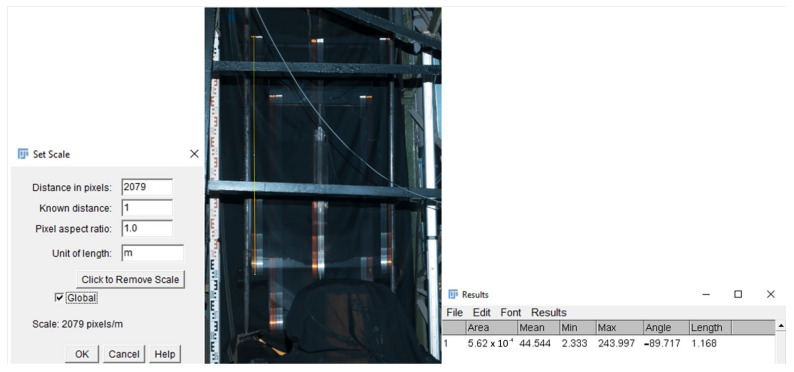
Scale coefficient settings.

The scale was compiled using the known lengths on a levelling staff and verified using the measurements of the known distances between the targets. The lengths were measured from the top, stationary position. The heights were measured using the open-source on-screen measurement software, ImageJ [38]. Results of the measurements were confirmed using the alternative freeware software, IC Measure [39]. Spatial calibration is available to provide real scale dimensional measurements in units, for example, millimetres. We used a scaling tool for geometric transformation. The software facilitated the measurement calibration (scale setting) based on a known length displayed in the image and by assigning a length in pixels in the image (Figure 8 on the left). The result of such calibration was a scaling coefficient, by which each distance measured in the image was multiplied while using the following equation:

(2) d=k·n,

where *d* is the measured length (height) (mm); *k* is the scale coefficient; and *n* is the number of pixels (mm).

The measurement could be carried out either as a measurement of a distance between the points visible in the image or directly as a length of the line. The accuracy of the measurement of these points depended on the accuracy of their visualisation in the image. The best results at a pixel level could be achieved by significantly zooming in the image. The measured lengths (heights) between the turning points were then expressed in predefined units, i.e., meters (Figure 8 in the middle and on the right). Finally, a scaling coefficient was determined for each image separately. 

#### 2.3.2. Distance Sensor Verification

**Figure 9:**
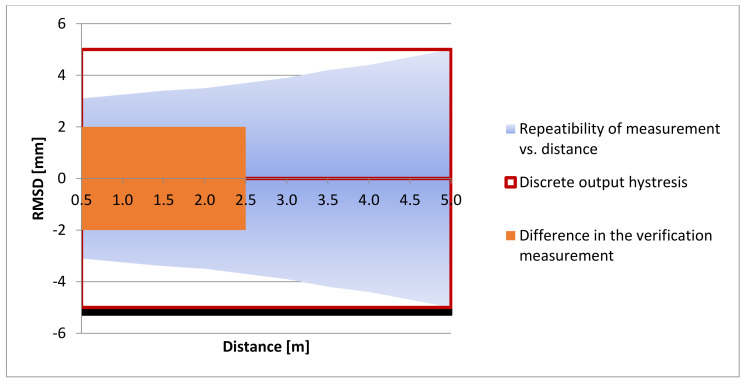
Verification of the L GAGE LT3 distance sensor accuracy.

The basic parameters of this instrument are specified in Section 2.2. According to the manufacturer’s data [40], the measurement accuracy parameters are expressed in two ways:

1. Discrete output hysteresis: 10 mm for a measurement with a frequency of 1 ms = 1000 Hz (a red line in Figure 9); or
2. Repeatability of measurement vs. distance: for a 1000 Hz record and a 90% white card (a blue area in Figure 9).

These values were verified with the impact hammer being in a stationary position at various heights of up to 2.5 m (on the horizontal axis in the graph in Figure 9). The comparison values were measured using the Leica TCRA 1201+ geodetic instrument with a GMP101 reflector prism with a constant of +17.5 mm. During the measurements, the instrument was placed on a geodetic tripod. The instrument accuracy is characterized by the accuracy of the measurement of angles m_ω_ = 1″ (0.3 mgon) and the accuracy of the measurement of lengths m_d_ = 1 mm + 1.5 ppm.

(3) $$m_{h}^{2}=\cos^{2}z.m_{S}^{2}+s^{2}.\sin^{2}z.{\left(\frac{m_{z}}{\rho }\right)}^{2},$$

where *m_h_* is the standard error of the height (mm); *s* is the slope distance (mm); *z* is the zenith angle (mgon); *m_z_* is the standard error of the measured zenith angle (mgon); and *m_s_* is the standard error of the slope distance (mm).

Considering the instrument accuracy parameters and the configuration of the experimental measurement, the accuracy of the measurement of an elevation between the measured positions was ±1 mm, as calculated using Equation (3). The verification measurement with the L-GAGE LT3 distance sensor was carried out as a trigonometric measurement of a height as the distance sensor was in a vertical position. Three independent verification measurements at each position were carried out. A graph in Figure 9 shows the resulting average values, i.e., up to ±2 mm (a red area in Figure 9). The dispersion of the differences in the verification measurements within the applied measurement range of up to 2.5 m were located within the interval of repeatability of measurement (a blue area in Figure 9). Therefore, the measured values corresponded to the data presented in the manufacturer’s documentation.

## 3. Results

Experimental measurements were carried out with the following objectives:

- To verify an alternative method for measuring a trajectory of an impact hammer in practical testing of conveyor belts;
- To identify the accuracy and reliability of the new method and compare them to the existing method.

**Figure 10:**
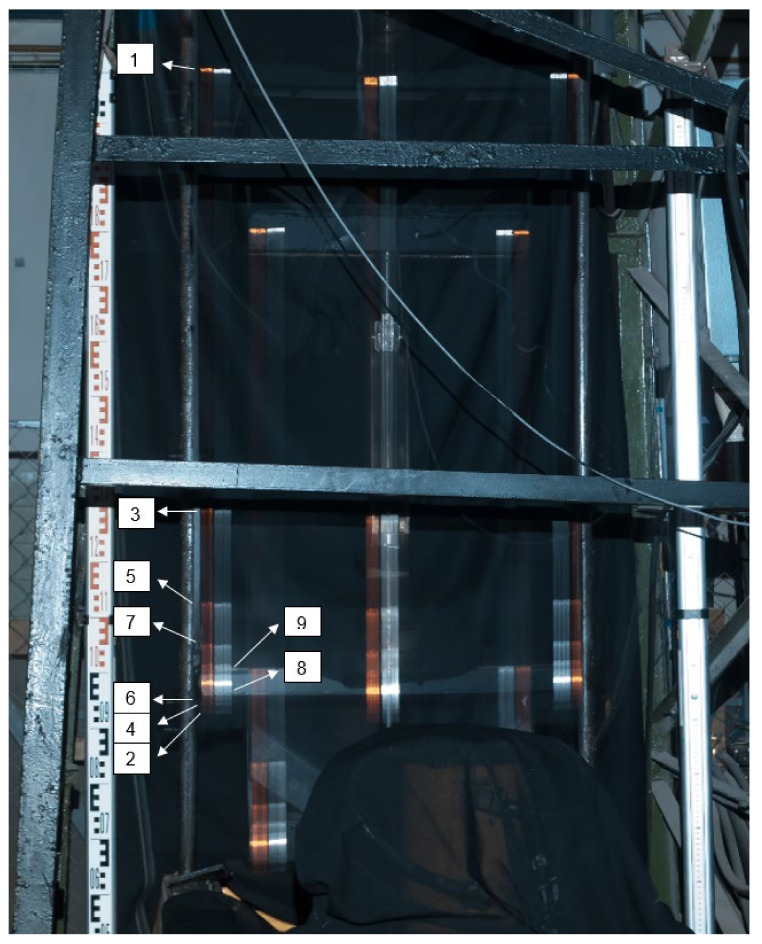
Impactor motion turning points.

Figure 10 shows an image with the impactor motion turning points. The other targets and images were evaluated using the same scheme. The bottom and top dead centres of the impactor motion turning were alternating.

Before further processing, the images were subjected to spectrometric adjustments to increase the image’s sharpness and highlight turning points in the impact hammer motion. The chapter which describes the results also contains the description of three independent repeated measurements and the evaluations of the experiment with identical configurations.

**Figure 11:**
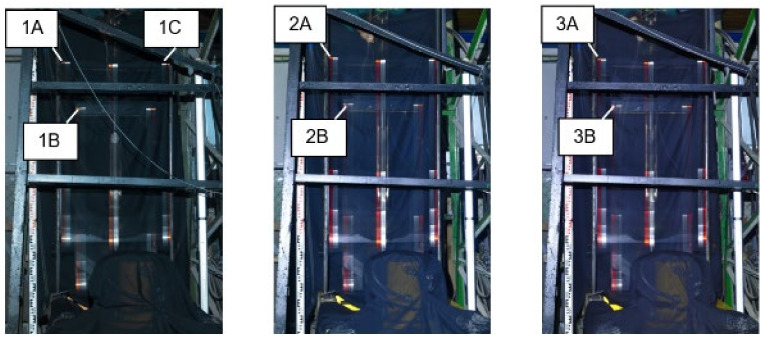
Images of Measurements; Measurement 1 with traces of reflective marks 1A, 1B, 1C on the left; Measurement 2 with traces of reflective marks 2A, 2B in the middle; and Measurement 3 with traces of reflective marks 3A, 3B on the right.

Figure 11 shows the images of the impact hammer motion captured in three experiments. The highlighted reflection marks were evaluated. The targets used in the individual experiments are designated as A, B, and C.

**Figure 12:**
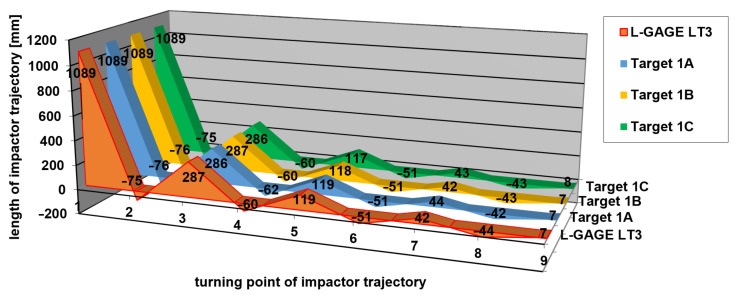
Measurement 1—relative heights of turning points.

**Figure 13:**
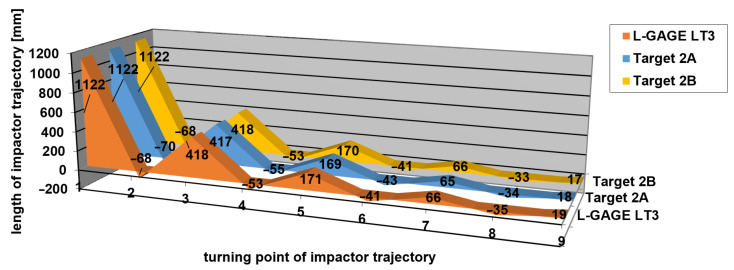
Measurement 2—relative heights of turning points.

**Figure 14:**
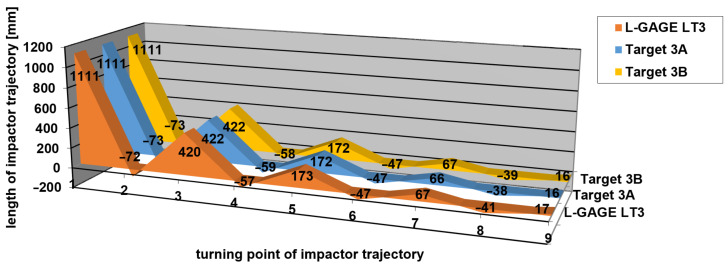
Measurement 3—relative heights of turning points.

Table 1, Table 2 and Table 3 contain the measured values of impact hammer heights in the turning points of its trajectory and the differences from a reference measurement carried out using the L-GAGE LT3 laser distance sensor according to Equation (4). A graphical representation of measured heights of the turning points is shown in Figure 12, Figure 13 and Figure 14, respectively. Height 0 corresponds to a height of a stretched belt in a resting horizontal position. The vertical axis shows the heights of the turning points in impact hammer motion, while the values on the horizontal axis correspond to the turning points in chronological order.

(4) $$\varepsilon_{i}=h_{i,REF}-h_{i,MEAS}.$$

**Figure 15:**
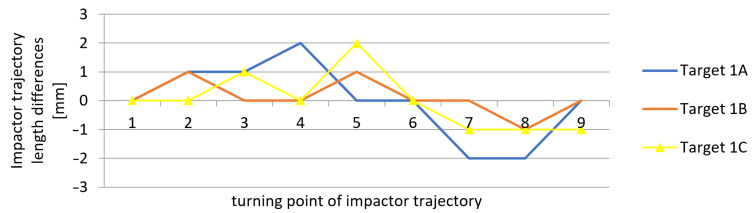
Measurement 1—differences in measured heights when compared to the reference height.

**Figure 16:**
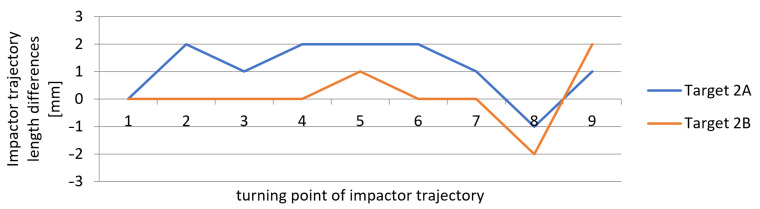
Measurement 2—differences in measured heights when compared to the reference height.

**Figure 17:**
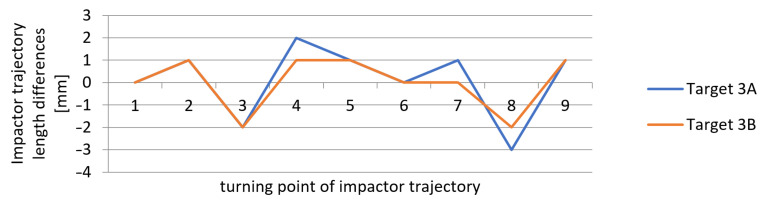
Measurement 3—differences in measured heights when compared to the reference height.

The differences between the values measured by applying the new measurement method and those obtained by the reference method (using a laser distance sensor), which resulted from Table 1, Table 2 and Table 3, are shown in Figure 15, Figure 16 and Figure 17. The vertical axis contains the observed differences, while the values on the horizontal axis correspond to the turning points in chronological order.

The results indicate that the differences between the values obtained by the new method and by the reference method represented ±2 mm, and in one of the measurements −3 mm. These values correspond to the theoretical accuracy of the measurements performed using the L-GAGE LT3 distance sensor in the dynamic mode with the recording frequency of 1000 Hz and to the values verified geodetically in the static mode. The average standard deviation of the observed differences from the reference measurement was up to ±1 mm. The measurements on multiple marks could verify the accuracy of the obtained results during the same experiment. We assumed that all of the marks on the impact hammer body travelled along the same trajectory. The correlation coefficient reached the value of 0.999 in all measurements for experiments 1 to 3.

## 4. Discussion

**Figure 18:**
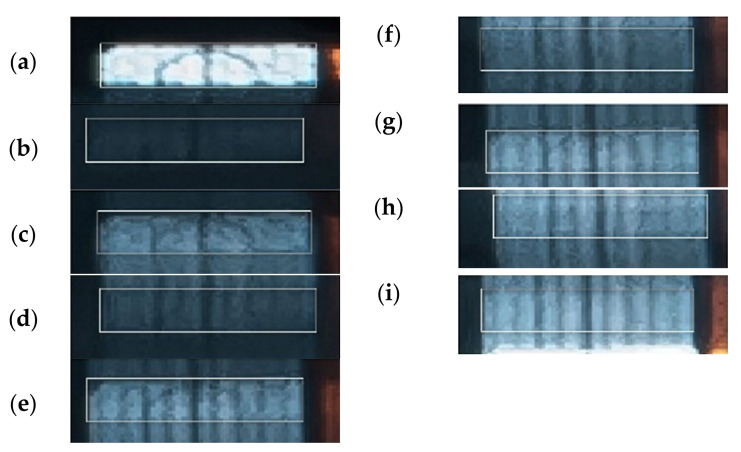
Image fragments showing the turning points of the impactor motion in Measurement 1 (photo) ((**a**,**c**,**e**,**g**,**i**) are the top turning points of the impactor motion; while (**b**,**d**,**f**,**h**) are the bottom turning points of the impactor motion, i.e., the bounces from the belt).

The accuracy of photographing the position of a turning point in the image is a decisive factor in assessing the reliability of the measurement results. Figure 18 shows the fragments of the images representing the turning points of the impactor motion. The coordinates (points in the image) were photographed manually. The same rectangle was used in each case; it was constructed with the dimensions of a respective target. The reference position was the position of its centre of gravity. The accuracy of the frame position was assumed to be approximately 2–3 pixels; this corresponded to the error of up to 1.5 mm. Figure 18a shows the baseline status of the system in a resting position. It corresponds to Turning Point 1. This image shows the reflection target mark in the highest quality. A rectangular shape was drawn with a size of 60 × 16 pixels; this corresponded to the dimensions of 30 × 10 mm. This frame was further used as a reference for bordering and the best possible photography of all the remaining turning points.

Figure 18b shows Turning Point 2, when the impactor bounced off. The time of endurance in this position is the shortest, as it was the first bounce from the tested belt, which means that the speed of the impact and the subsequent bounce was the highest. This image exhibited the lowest brightness value—it was the least clear, but still well-read and clearly bordered image. Figure 18c,e,g,i show the top turning points of the impactor motion, while Figure 18d,f,h show the bottom turning points of the impactor motion when it bounced back from the belt.

The amplitudes of subsequent bounces gradually approached the zero value. The obtained image seems to be continuous due to the repeated motion of the reflection target. Another affecting factor is the lower and more even speed of the impactor motion. As a result, turning points were gradually less identifiable. Turning points representing the bounces from the belt were always less clear. They are displayed in Figure 18b,d–f. Figure 8i shows the last turning point, Turning Point 9.

A subsequent error, caused by poorer identification of the target in the image, ranged from 3 to 4 pixels; in reality, this corresponds to 2 mm.

In the performed experiment, these nine turning points seem to be the impactor’s maximum possible number of detectable bounces. This corresponds to the last bounce-off at the height of 10 through 20 mm. However, the subsequent bounces could not be reliably identified, as the motion trail was not sufficiently clear.

## 5. Conclusions and Future Work

The experiments’ analysis and evaluation indicated good accordance with the trajectory of the impact hammer measured by applying the novel measurement method compared to the currently used and verified measuring instrument. The standard deviation identified in all the experiments was 1 mm.

Advantages of the novel method: low costs; no need for special hardware (distance sensor); the measurement speed and constant measurement accuracy along the entire impactor trajectory (in a measurement carried out using a distance sensor, the accuracy decreases with an increasing distance).

Disadvantages of the novel method: the necessity of specific illumination conditions; data are only obtained after the post-processing; only the turning points may be identified, not the entire course of the impact hammer motion; some turnovers cannot be evaluated due to overlapping or blur; and not every experiment is successful.

In terms of future work, our experimental research presented in this article was performed in order to verify the feasibility and accuracy of the proposed method under selected conditions. The parameters of the initial height and weight of the impactor, which are commonly used in laboratory tests of belts, were chosen. The belt testing device also allows approximately twice the initial height of the hammer. Therefore, we will expand the testing of our new measurement method with the ranging value of height and weight of the impactor in our future research. The basic implementation premise is to increase the distance of the camera from the measuring object. However, resulting is a change in the source and intensity of illumination so that it is possible to simultaneously identify the trace of reflective marks on the images and at the same time values on the scales determining the scale factor. The assessment of our novel method also led us to the idea of using a camera with high-speed recording. In future research, after the adaptation of the spatial arrangement of the conveyor belt test device, we plan to use this method of measuring the deflection of the belts and the turning points of the hammer. The results will be compared with the reference and our proposed method. A separate part of the future research will be the mathematical description of our novel approach and the creation of a mathematical model to derive parameters for computer simulation for testing samples of the conveyor belts.

The proposed method allows the identification of the turning points of a trajectory of the impact hammer on a testing stand without identifying the time data. Future work will also focus on the design, construction, and verification of a measuring device capable of identifying a trajectory of the impactor and the time. The underlying principle will be similar to the principle of the method described above, but it will be supplemented with active illumination of the impactor and a rotary element in the head of the camera tripod. It can record the rotation of a camera with an open shutter during a measurement. We assume that the obtained images will enable the identification of the time series of the impacts of an impactor in the performed experiments.

The obtained results relate to the accuracy of the currently used measuring instrument. Therefore, we regard the novel method for measuring belt deflection in laboratory tests as a suitable alternative or a verification (calibration) procedure to be applied together with the currently used measurement methodology.

## Figures and Tables

**Table 1:** Results of the measurements of the trajectory in turning points—Measurement 1.

Turning Point	Reference Measurement (L-GAGE LT3) (mm)	Top Left 1A (mm)	Bottom Left 1B (mm)	Top Right 1C (mm)	Differences Ref -1A (mm)	Differences Ref -1B (mm)	Differences Ref -1C (mm)
1	1089	1089	1089	1089	0	0	0
2	−75	−76	−76	−75	1	1	0
3	287	286	287	286	1	0	1
4	−60	−62	−60	−60	2	0	0
5	119	119	118	117	0	1	2
6	−51	−51	−51	−51	0	0	0
7	42	44	42	43	−2	0	−1
8	−44	−42	−43	−43	−2	−1	−1
9	7	7	7	8	0	0	−1

**Table 2:** Results of the measurement of the trajectory in turning points—Measurement 2.

Turning Point	Reference Measurement (L-GAGE LT3) (mm)	Top Left 2A (mm)	Bottom Left 2B (mm)	Differences Ref -2A (mm)	Differences Ref -2B (mm)
1	1122	1122	1122	0	0
2	−68	−70	−68	2	0
3	418	417	418	1	0
4	−53	−55	−53	2	0
5	171	169	170	2	1
6	−41	−43	−41	2	0
7	66	65	66	1	0
8	−35	−34	−33	−1	−2
9	19	18	17	1	2

**Table 3:** Results of the measurements of the trajectory in turning points—Measurement 3.

Turning Point	Reference Measurement (L-GAGE LT3) (mm)	Top Left 3A (mm)	Bottom Left 3B (mm)	Differences Ref-3A (mm)	Differences Ref-3B (mm)
1	1111	1111	1111	0	0
2	−72	−73	−73	1	1
3	420	422	422	−2	−2
4	−57	−59	−58	2	1
5	173	172	172	1	1
6	−47	−47	−47	0	0
7	67	66	67	1	0
8	−41	−38	−39	−3	−2
9	17	16	16	1	1
